# Fetal Bovine Hide Collagen–Chitosan Composite Sponges: Preparation, Physicochemical Profiling, and Cutaneous Wound Healing Efficacy

**DOI:** 10.3390/polym18182198

**Published:** 2026-09-09

**Authors:** Linying Ni, Xinxing Zheng, Ling Du, Wenjing Mu, Xin Wang, Yongming Zhang

**Affiliations:** 1 College of Life Science and Technology, Inner Mongolia Normal University, Hohhot 010022, China; 20234015009@mails.imnu.edu.cn (L.N.); 20250009@imnu.edu.cn (X.Z.); nmduling@imnu.edu.cn (L.D.); muwenjing@imnu.edu.cn (W.M.); 2 Key Laboratory of Biodiversity Conservation and Sustainable Utilization in Mongolian Plateau for College and University of Inner Mongolia Autonomous Region, Hohhot 010022, China; 3 Inner Mongolia Daxi Biotechnology Co., Ltd., Hohhot 010000, China; m13674850655@163.com

**Keywords:** fetal bovine hide, agricultural byproduct, collagen, sponge, chitosan, wound healing

## Abstract

The escalating production of fetal bovine serum generates substantial quantities of fetal bovine hide as an underutilized byproduct. In this study, we extracted collagen from this source, characterized it as predominantly type I collagen with intact triple-helical features, and fabricated a series of composite sponge dressings by blending it with chitosan. The best-balanced formulation (COL_1_/CS_1_, 1:1 ratio) exhibited markedly superior physicochemical properties relative to pure collagen sponges, as evidenced by higher porosity (91.3%), water uptake (2010%), moisture retention (23.7%), and water vapor transmission rate (4169.02 ± 86.45 g/m^2^/day). We hypothesize that the intrinsically lower cross-linking density of fetal collagen may expose a greater abundance of carboxyl and hydroxyl moieties, thereby fostering electrostatic complexation and hydrogen bonding with chitosan’s amino groups. This molecular interplay appears to promote the genesis of a highly uniform, interconnective porous network. In vitro, the COL_1_/CS_1_ sponge elicited a hemolysis rate below 5%, a blood coagulation index as low as 7.02%, no cytotoxicity toward L929 and MRC-5 cells, and a pronounced capacity to stimulate cell proliferation and wound repopulation. In a murine full-thickness excisional wound model, the COL_1_/CS_1_ group achieved a 98.1% closure rate by day 14, significantly outpacing both the pure collagen and untreated controls. Histological examinations corroborated these findings, revealing accelerated granulation tissue deposition, robust neovascularization, and orderly collagen remodeling, with no overt toxicity observed in vital organs under the tested conditions. This work presents a viable valorization pathway for an agricultural byproduct into high-value biomedical constructs and provides insights into how source-dependent collagen attributes may influence the functional performance of biomaterials.

## 1. Introduction

The burgeoning industrialization of stem cell therapies [1] and gene-engineered biologics [2] has propelled global demand for fetal bovine serum (FBS) [3]. This reliance on fetal calves for serum production inevitably yields a massive annual volume of fetal bovine hide as a processing coproduct [4]. As a derivative of both agricultural and biomedical industries, fetal bovine hide confers distinct advantages over conventional collagen sources—such as adult bovine hide, bovine tendon, porcine dermis, or fish skin [5]. First, the intrauterine environment effectively shields the fetus from exogenous antigens and environmental toxins, resulting in skin with an inherently lower antigenic burden. Second, the centralized nature of FBS production lines ensures minimal batch-to-batch variability, offering exceptional feedstock consistency. Third, compared to mature tissues, fetal bovine skin exhibits a diminished degree of intermolecular cross-linking, which not only improves its solubility and processability but also enhances the accessibility of bioactive cell-binding motifs [6]. Furthermore, fetal-derived materials intrinsically carry a reduced risk of prion or zoonotic pathogen transmission. Collectively, these inherent attributes position fetal bovine hide as a premium collagen source, distinguished by suggested low immunogenicity, outstanding homogeneity, and favorable bioactivity, thereby offering a compelling opportunity to upcycle this agricultural residue into sophisticated medical materials.

Collagen (COL), the predominant structural protein of the extracellular matrix, finds extensive biomedical application owing to its unique triple-helical conformation, characteristic hydroxyproline residues, low immunogenicity, excellent biocompatibility, and controllable biodegradability [7,8,9]. Beyond its structural functions, type I collagen orchestrates profound biological effects via specific molecular recognition events. The GFOGER (Gly-Phe-Hyp-Gly-Glu-Arg) sequence embedded within the triple helix serves as a high-affinity binding motif for integrins α_1_β_1_ and α_2_β_1_, which are ubiquitously expressed on fibroblasts, platelets, vascular cells, and epithelial cells [10]. Integrin-mediated adhesion to collagen not only anchors cells to the matrix but also triggers downstream signaling cascades governing proliferation, migration, differentiation, and survival [11]. In the context of wound repair, these integrin–collagen interactions are critical for dynamic connective tissue remodeling, coordinating fibroblast activation, myofibroblast differentiation, and the ultimate restoration of tissue architecture [12]. Consequently, preserving the native triple-helical integrity and GFOGER-containing sequences is paramount for harnessing the full biological potential of collagen-based materials—rendering it an indispensable building block for hemostatic agents [13], tissue engineering scaffolds [14,15], and wound dressings [16,17].

Cutaneous wound healing progresses through four overlapping yet distinct phases: hemostasis, inflammation, proliferation, and remodeling [18]. An ideal wound dressing must be multifunctional, catering to the specific demands of each phase: swiftly absorbing exudate and activating coagulation during hemostasis; providing a three-dimensional porous template for cellular ingress and proliferation during the proliferative phase; and sustaining an optimally moist microenvironment throughout the healing trajectory [19,20]. Collagen sponges, characterized by their highly interconnected porous architecture and extensive specific surface area, are well-suited to meet these criteria. They efficiently absorb wound exudate and furnish an ideal interface for platelet adhesion and activation, thereby accelerating hemostasis [21]. Moreover, the fibrillar ultrastructure of collagen sponges mimics the native extracellular matrix topography, promoting fibroblast polarization and directional migration through contact guidance [22]. The elevated surface area also facilitates the sequestration and concentration of endogenous growth factors, such as VEGF and PDGF, fostering a microenvironment conducive to angiogenesis and tissue regeneration [23].

Chitosan, a naturally derived polysaccharide obtained via partial deacetylation of chitin, possesses well-documented hemostatic, antibacterial, and pro-healing activities [24]. It accelerates granulation tissue maturation, promotes re-epithelialization, and modulates macrophage polarization toward a pro-regenerative M2 phenotype [25]. Composite sponges combining collagen and chitosan synergistically integrate these complementary properties, merging the cell-recognition functions of collagen with the pro-healing attributes of chitosan [23,24,26]. At the molecular level, this synergy is rooted in specific intermolecular interactions. Under the weakly acidic to neutral conditions typical of composite fabrication, the amino groups (-NH_3_^+^) of chitosan form electrostatic complexes with the carboxylate groups (-COO^−^) of collagen, while concurrent intermolecular hydrogen bonds form between chitosan’s hydroxyl groups and collagen’s carbonyl, amide, and hydroxyl moieties [27,28]. FTIR and 2D correlation analyses have confirmed these multimodal interactions, revealing that chitosan incorporation can partially unwind the collagen triple helix, exposing previously sequestered functional groups [28]. This molecular crosstalk curbs excessive collagen fibril aggregation, promoting a more uniform pore architecture relative to pure collagen sponges [29]. Such an optimized microstructure is critical for efficient nutrient diffusion and metabolic waste removal, thereby sustaining cell viability and proliferation throughout the scaffold’s entire thickness [30].

Crucially, collagens from disparate sources exhibit variations in primary structure, cross-linking density, amino acid composition, and post-translational modifications—factors that profoundly influence their physicochemical traits and biological performance [14]. For instance, interspecies and inter-tissue differences in amino acid profiles and cross-linking patterns impact thermal stability, degradation kinetics, solubility, and integrin-binding affinity [31,32]. Age-related cross-linking further alters collagen’s hierarchical organization and its susceptibility to enzymatic breakdown [31]. Given these source-dependent structural nuances, the precise molecular interactions between collagen and chitosan are likely to vary considerably. Fetal bovine hide collagen, characterized by lower intermolecular cross-linking density and reduced molecular ordering, differs in its surface-exposed functional groups from collagen derived from adult bovine Achilles tendon or porcine skin [6]. How these unique structural attributes modulate electrostatic and hydrogen-bonding interactions with chitosan, and how they ultimately influence the pore architecture and biological performance of the composite sponges, has remained largely unexplored. Most existing investigations on collagen–chitosan composites have relied on conventional sources, such as bovine tendon, porcine dermis, or fish skin [33,34], leaving the systematic application of fetal bovine hide collagen within this composite system unaddressed.

Previous work has demonstrated the successful extraction of structurally intact type I collagen from fetal bovine hide, with preliminary characterization confirming its molecular weight distribution, amino acid profile, and thermal stability, thereby validating its viability as a collagen source [6]. However, no study to date has systematically evaluated the physicochemical properties and wound healing potential of composite sponges fabricated from fetal bovine hide collagen and chitosan. In this contribution, we extracted type I collagen from fetal bovine hide, prepared a series of collagen–chitosan composite sponges with varying ratios, identified the best-balanced formulation through comprehensive physicochemical characterization, and rigorously assessed its pro-healing efficacy using in vitro cellular assays and an in vivo murine full-thickness skin defect model. This work provides a practical technological pathway for valorizing a major agricultural byproduct of the FBS industry, expands the repertoire of collagen sources for wound dressing applications, and offers insights into the possible source-dependent interactions between collagen and chitosan. The limitation is that this study did not compare with the source of adult bovine collagen and did not provide direct molecular evidence for the proposed signaling pathway.

## 2. Materials and Methods

### 2.1. Extraction of Collagen from Fetal Bovine Hide

Fetal bovine hides were procured from Inner Mongolia Daxi Biotechnology Co., Ltd. (Hohhot, China). The hides were depilated, meticulously scraped to remove internal impurities, thoroughly washed with ultrapure water, and cut into 5 × 5 cm pieces. These pieces were then centrifuged at 8000 rpm for 20 min in a high-speed refrigerated centrifuge (H17.6R, Shanghai Luxiangyi Centrifuge Instrument Co., Ltd., Shanghai, China) to remove excess water. Defatting was performed by soaking the pieces in 5% Na_2_CO_3_ solution at a solid-to-liquid ratio of 1:10 (*w*/*v*) under continuous stirring for 24 h. Following defatting, the pieces were rinsed with ultrapure water until the washings were neutral or slightly alkaline. Subsequently, the pieces were soaked in 2% NaCl solution at a ratio of 1:10 (*w*/*v*) for 24 h under stirring to eliminate salt-soluble impurities, then rinsed with ultrapure water and centrifuged again at 8000 rpm for 20 min. During both defatting and impurity-removal steps, the soaking solutions were replaced every 12 h.

Acid-soluble collagen (ASC) was extracted following a modified protocol of Indriani et al. [35]. Briefly, the pretreated hide pieces were soaked in 0.5 M acetic acid at a solid-to-liquid ratio of 1:10 (*w*/*v*) until swollen and translucent. The swollen tissues were then homogenized, and additional 0.5 M acetic acid was added to achieve a final ratio of 1:30 (*w*/*v*). The mixture was stirred for 48 h for extraction. The crude extract was filtered through gauze, and the filtrate was centrifuged at 10,000 rpm for 20 min. The supernatant was collected, and NaCl was added to a final concentration of 0.9 M to salt out the collagen overnight. After centrifugation at 10,000 rpm for 20 min, the precipitate was redissolved in 0.5 M acetic acid. The solution was dialyzed against 0.1 M acetic acid for 48 h (with AgNO_3_ testing confirming no Cl^−^ precipitation), followed by dialysis against ultrapure water for another 48 h until neutrality. Finally, the dialysate was lyophilized using a vacuum freeze dryer (GIPP-02FDA, Shanghai Jipu Electronic Technology Co., Ltd., Shanghai, China) to obtain ASC. For pepsin-soluble collagen (PSC), the procedure was identical except that pepsin (2% of the tissue weight) was added to the acetic acid during extraction. All steps were performed at 4 °C.

The collagen extraction yield was calculated based on hydroxyproline content, using a conversion factor of 7.1 for terrestrial mammals, with the following equations:

(1) Collagen content=hydroxyproline content×7.1

(2) $$Extraction\;yield\;\left(\%\right)=\frac{Collagen\;content\;in\;the\;extract}{Collagen\;content\;in\;the\;pretreated\;skin}\times 100\%$$

### 2.2. Characterization of Fetal Bovine Hide Collagen

#### 2.2.1. SDS-PAGE

ASC and PSC were dissolved in 0.5 M acetic acid to prepare 1 mg/mL collagen solutions. Each protein solution was mixed with 5× loading buffer at a volume ratio of 4:1, heated at 100 °C for 5 min, and cooled on ice. Samples (10 µL) and a pre-stained protein marker (5 µL) were loaded onto a gel consisting of 6% separating gel and 5% stacking gel. After electrophoresis, the gel was stained with Coomassie Brilliant Blue R-250 staining solution (0.1% *w*/*v* R-250, 45% *v*/*v* methanol, 10% *v*/*v* glacial acetic acid) for 30 min, and then destained with destaining solution (12% *v*/*v* methanol, 7% *v*/*v* glacial acetic acid) until clear bands appeared. The gel was imaged using a gel documentation system (Universal Hood II, Bio-Rad Laboratories, Shanghai, China).

#### 2.2.2. UV-Vis Spectroscopy

UV-visible spectra of ASC and PSC (1 mg/mL in 0.5 M acetic acid) were recorded on a UV-2600i Plus spectrophotometer (Shimadzu, Kyoto, Japan) over the wavelength range of 200–400 nm at a scan speed of 2 nm/s, using 0.5 M acetic acid as the blank.

#### 2.2.3. Fourier Transform Infrared (FTIR) Spectroscopy

Lyophilized ASC and PSC samples were placed on a clean sample stage, and FTIR spectra were acquired using a Nicolet iS50 spectrometer (Thermo Fisher Scientific, Waltham, MA, USA) in the range of 4000–500 cm^−1^ with a resolution of 2 cm^−1^. For secondary structure analysis, the amide I region (1600–1700 cm^−1^) was deconvoluted and fitted using PeakFit version 4.12 software with Gaussian curve fitting (R^2^ > 0.99).

#### 2.2.4. Scanning Electron Microscopy (SEM)

Small pieces of ASC and PSC were mounted on stubs, sputter-coated with gold, and examined under a field-emission SEM (Gemini SEM 300, Carl Zeiss, Oberkochen, Germany) at an accelerating voltage of 5 kV to observe and capture microstructural images.

### 2.3. Preparation of COL/CS Composite Sponges

PSC and chitosan were each dissolved in 1% acetic acid to prepare 1% (*w*/*v*) collagen solution (COL) and 1% (*w*/*v*) chitosan solution (CS). The two solutions were mixed according to the ratios listed in Table 1, and the pH was adjusted to neutral with NaOH. Then, 2 mL of each mixture was dispensed into 12-well plates and allowed to stand at room temperature for 30 min to permit self-assembly. The plates were then lyophilized to obtain COL/CS composite sponges. All sponges were sterilized under UV irradiation for 24 h and stored at low temperature in sealed containers.

### 2.4. Physicochemical Characterization of COL/CS Composite Sponges

#### 2.4.1. Apparent Density

Following the method of Yang et al. [21], the mass of the collagen sponge was weighed, and the diameter and thickness of the sponge samples were measured with a vernier caliper. The apparent density was then calculated according to the formula.

(3) $$\rho \left(mg/cm^{3}\right)=\frac{m}{\pi {\left(D/2\right)}^{2}h}$$

Note: *ρ* denotes density, π is taken as 3.14, D is the diameter, and h is the thickness.

#### 2.4.2. Porosity

Porosity was determined by the ethanol displacement method described by Tang et al. [36]. Briefly, a pre-weighed sponge (m) was placed in a centrifuge tube containing a known volume of absolute ethanol. The total mass (m_1_) of the tube plus ethanol was recorded. After the sponge was fully immersed for 30 min, the ethanol level was adjusted to the mark, and the mass (m_2_) was measured. The sponge was then removed, and the mass of the remaining ethanol with the tube (m_3_) was recorded. Porosity was calculated as:

(4) $$Porosity\left(\%\right)=\frac{m_{1}-m_{3}-m}{m_{1}-m_{3}}\times 100\%$$

#### 2.4.3. Water Absorption

Following the method of Lin et al. [37] with modifications, pre-weighed sponges (W_0_) were immersed in ultrapure water for 2 h to allow swelling. After removal, the excess surface water was drained for 1 min, and the wet weight (W_1_) was measured. Water absorption (WA) was calculated as:

(5) $$W\left(\%\right)=\frac{W_{2}-W_{1}}{W_{1}}\times 100\%$$

#### 2.4.4. Moisture Retention

Moisture retention was assessed according to Sun et al. [31]. Swollen sponges were centrifuged at 500 rpm for 3 min and then weighed (W). Moisture retention (WR) was expressed as:

(6) $$Water\;retention\left(\%\right)=\frac{W-W_{\mathrm{dry}}}{W_{\mathrm{dry}}}$$

Note: WR denotes the moisture retention of the sponge sample, W is the mass after centrifugation, and W_dry_ is the mass of the dried sample.

#### 2.4.5. Water Vapor Transmission Rate (WVTR)

The water vapor transmission method of Wang et al. [38] was adopted. Sponge samples were placed over the mouths of centrifuge tubes containing 9 mL of ultrapure water. An open tube served as the control. The initial weights were recorded. After 24 h at 37 °C, the final weights were measured. WVTR was calculated as:

(7) $$WVTR\left(g/m^{2}/day\right)=\frac{W_{i}-W_{t}}{S}$$

Note: where S, W_i_, and W_t_ are the bottle mouth area (m^2^), bottle weight (g), and the bottle weight (g) after 24 h, respectively.

### 2.5. In Vitro Biological Evaluation

#### 2.5.1. Preparation of Extracts

Extracts were prepared according to GB/T 16886.12-2017 (Biological evaluation of medical devices–Part 12: Sample preparation and reference materials). COL and COL_1_/CS_1_ sponges (0.5–1.0 mm thickness) were immersed in extraction media at a ratio of 1.25 cm^2^/mL: normal saline for hemolysis tests, and serum-free MEM medium (supplemented with 1% Penicillin-Streptomycin) for cell-based assays. Extraction was performed at 37 °C for 24 h with gentle shaking.

#### 2.5.2. Hemolysis Test

Hemolysis was evaluated following YY/T 1651.1-2019 with minor modifications. Rabbit anticoagulated whole blood was diluted with normal saline (SC) at a volume ratio of 4:5. Negative control tubes received 5 mL SC, positive control tubes received 5 mL distilled water (DW), blank tubes received 5 mL diluted blood (NS), and experimental groups (COL and COL_1_/CS_1_) received 5 mL of their respective saline extracts. All tubes were incubated at 37 °C for 30 min, then 0.1 mL of diluted blood was added to each tube except the blank, followed by gentle mixing. After further incubation at 37 °C for 60 min (with gentle inversion every 30 min), the contents were transferred to fresh tubes and centrifuged at 800× *g* for 5 min. The supernatant absorbance at 545 nm was measured [32]. Hemolysis ratio (HR) was calculated as:

(8) $$Hemolysis\;rate(\%)=\frac{A-B}{C-B}\times 100\%$$

Note: A is the absorbance of the test group, B is that of the negative control, and C is that of the positive control.

#### 2.5.3. Blood Coagulation Index (BCI) Assay

This assay was adapted from Jiang et al. [33]. Equal masses of commercial gauze and composite sponges were placed in Petri dishes. Rabbit anticoagulated whole blood (200 µL) was slowly dropped onto each sample, followed by 20 µL of 0.2 M CaCl_2_ to initiate coagulation. After 5 min, 25 mL of ultrapure water was added, and the dishes were incubated at 37 °C for 5 min with gentle shaking. The supernatant was collected, and its absorbance at 540 nm was measured. As a reference, 0.1 mL of anticoagulated blood was diluted with ultrapure water to 25 mL, and its absorbance was taken as 100%. BCI was calculated as:

(9) $$BCI(\%)=\left(B_{t}/B_{0}\right)\times 100\%$$

Note: where B_t_ is the absorbance of the control and test groups, and B_0_ is the absorbance of the diluted blood.

#### 2.5.4. Cytotoxicity Assay

L929 cells (Wuhan Zishan Biotechnology Co., Ltd., Wuhan, China) and MRC-5 cells (Inner Mongolia Daxi Biotechnology Co., Ltd., Hohhot, China) were cultured in MEM medium (Gibco, Invitrogen, Carlsbad, CA, USA) supplemented with 10% FBS (Inner Mongolia Daxi Biotechnology Co., Ltd.) and 1% Pen-Strep at 37 °C in 5% CO_2_. Cells at passage 3 were used. L929 and MRC-5 cells were seeded in 96-well plates at densities of 3.0 × 10^4^ and 5.0 × 10^4^ cells/well, respectively. After 24 h, the medium was replaced with 100 µL of sponge extracts (MEM medium as a negative control, phenol solution as a positive control). After further incubation for 24 h (or 24, 48, and 72 h for proliferation assays), the extracts were removed, and 100 µL of serum-free medium containing 10% CCK-8 reagent (Beijing Solarbio Science & Technology Co., Ltd., Beijing, China) was added. Plates were incubated in the dark for 30–60 min, and absorbance at 450 nm was measured [32]. Cell viability was calculated according to the manufacturer’s instructions.

#### 2.5.5. Cell Proliferation Assay

Cell seeding and extract treatment were performed as in Section 2.5.4. At 24, 48, and 72 h, the medium was replaced with CCK-8 solution, and absorbance was read at 450 nm to directly reflect proliferation.

#### 2.5.6. Scratch Wound Healing Assay

L929 cells were seeded in 6-well plates at 2.0 × 10^5^ cells/well and cultured in complete MEM medium containing 10% FBS for 24 h to reach confluence. A sterile 200 µL pipette tip was used to create a vertical scratch across the monolayer. Debris was gently washed away with PBS. The cells were then incubated with 2 mL of sponge extracts prepared in serum-free MEM medium (supplemented with 1% Pen-Strep), while the control group received the same volume of serum-free MEM medium (supplemented with 1% Pen-Strep). Images were captured immediately (0 h) and at 24 h post-scratch using an inverted microscope at the same positions to record changes in scratch width, which were then measured using ImageJ 1.53t.

(10) $$\mathrm{Closure}\;\mathrm{rate}\left(\%\right)=\frac{\left(W_{0h}/W_{24h}\right)}{W_{0h}}\times 100\%$$

Note: W_0_ represents the scratch width at 0 h, and W_24_ represents the scratch width at 24 h.

### 2.6. In Vivo Wound Healing and Biosafety Evaluation

#### 2.6.1. Mouse Full Thickness Skin Defect Model

Thirty SPF female C57BL/6J mice (8 weeks old, 25 ± 2 g) were acclimatized for 7 days prior to the experiment. Animals were housed under standard conditions (12 h light/dark cycle, 22 ± 2 °C, 50 ± 10% humidity) with ad libitum access to food and water. Prior to surgery, mice were randomly divided into three groups (*n* = 10) using a computer-generated random number table: blank control (gauze), COL sponge, and COL_1_/CS_1_ sponge. After intraperitoneal anesthesia with sodium pentobarbital (50 mg/kg), the dorsal skin was shaved and disinfected. A full-thickness skin defect of approximately 8 mm × 8 mm was created on the back with surgical scissors. The respective sponges were applied directly to the wound, covered with sterile gauze, and secured with adhesive tape to prevent gnawing and dislodgement. Dressings were changed, and photographs were taken on days 0, 3, 7, 10, and 14. For histological analysis, two mice from each group were sacrificed on days 3, 7, 10, and 14 post-surgery; the remaining animals at day 14 were used for major organ toxicity evaluation. No animals were excluded from the analysis.

#### 2.6.2. Wound Closure Rate

Wound areas at each time point were traced using ImageJ’s freehand tool, and the closure rate was calculated as:

(11) $$Wound\;closure\;rate\left(\%\right)=\frac{\left(A_{0}/A_{t}\right)}{A_{0}}\times 100\%$$

Note: where A_t_ is the wound area at a given time, and A_0_ is the wound area at day 0.

#### 2.6.3. Histological Evaluation

Mice were sacrificed on days 3, 7, 10, and 14 post-surgery. Full-thickness skin tissues, including the wound and surrounding margins, were harvested, fixed in 4% paraformaldehyde for 24 h, dehydrated through graded ethanol, cleared in xylene, and embedded in paraffin. Sections were stained with hematoxylin and eosin (H&E) and Masson’s trichrome for histological examination.

Semi-quantitative analysis of histological sections was performed using ImageJ software. Granulation tissue thickness was measured on H&E-stained sections at the central wound area, where three randomly selected fields per section were used to measure the vertical distance from the newly formed epidermal basal layer to the junction of granulation tissue and subcutaneous tissue; the average value was calculated and expressed in micrometers (μm). Collagen deposition was quantified on Masson-stained sections by selecting three random fields per section and calculating the percentage of blue-positive area relative to the total tissue area using ImageJ, expressed as collagen area fraction (%).

(12) $$Collagen\;area\;fraction\left(\%\right)=\frac{Blue-positive\;area}{Total\;tissue\;area}\times 100\%$$

#### 2.6.4. Biosafety Analysis

On day 14, heart, liver, spleen, lung, and kidney tissues were collected from each group, fixed, embedded, sectioned, and stained with H&E to evaluate potential organ toxicity.

### 2.7. Statistical Analysis

All data are expressed as mean ± standard deviation (SD). Statistical analyses were performed using Origin 2018 and GraphPad Prism 10. For in vitro material-related tests, the error bar is the standard error obtained by three independent measurements (*n* = 3), and the newly prepared sponge sample is used in each independent experiment. Comparisons between two groups were performed using a two-tailed Student‘s *t*-test. Comparisons among multiple groups were analyzed by one-way analysis of variance (ANOVA) with Tukey’s post hoc test. Data involving both group and time factors were evaluated using two-way repeated-measures ANOVA with Tukey’s post hoc test. *p* < 0.05 was considered statistically significant (* *p* < 0.05, ** *p* < 0.01, *** *p* < 0.001, **** *p* < 0.0001).

## 3. Results and Discussion

### 3.1. Collagen Extraction Yield

**Figure 1:**
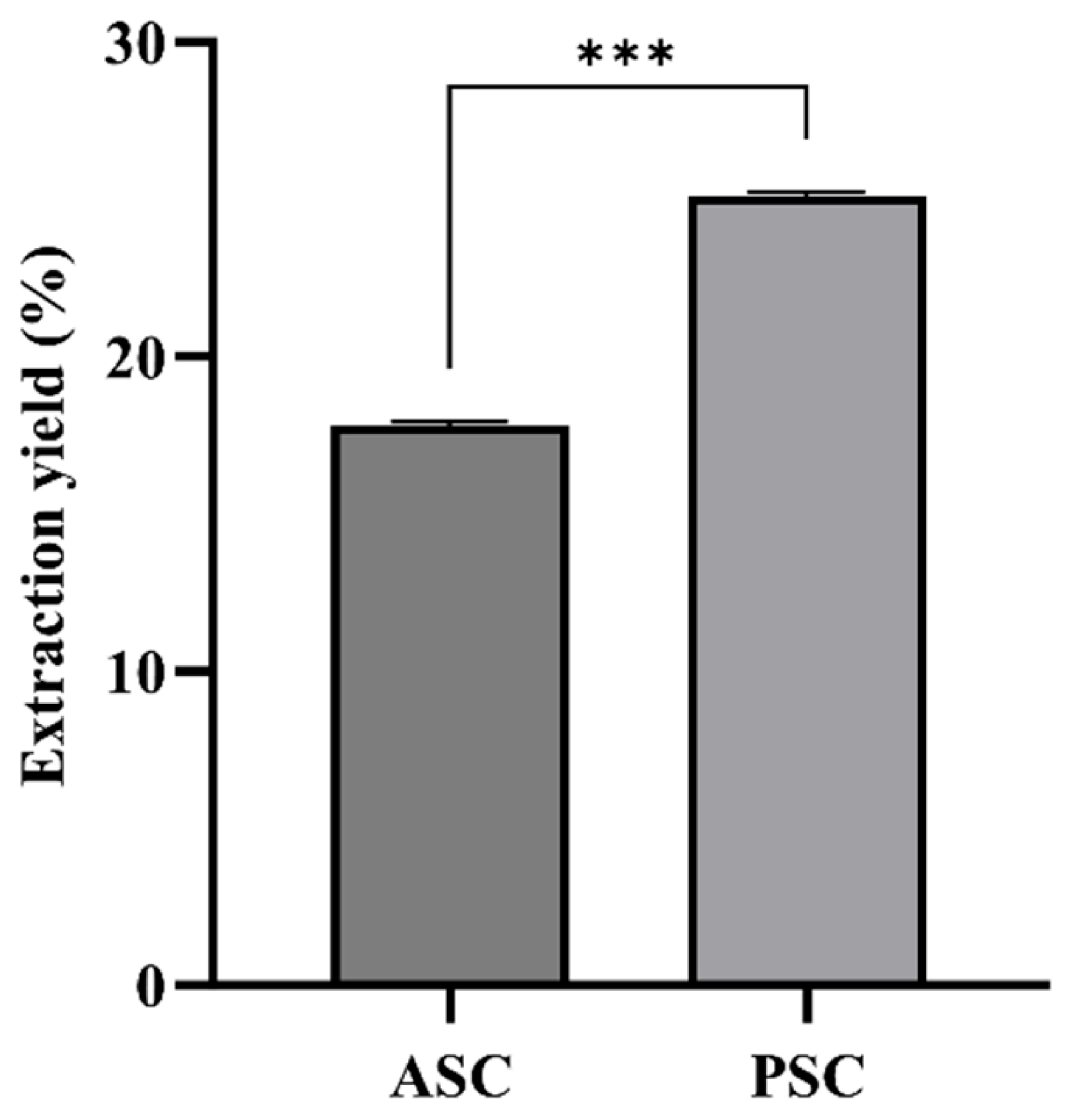
Effects of different extraction methods on the extraction rate of collagen from fetal bovine skin (*n* = 3, *** means *p* < 0.001).

The extraction yields of ASC and PSC from fetal bovine hide were 17.81 ± 0.12% and 25.08 ± 0.16%, respectively, with PSC showing a significantly higher yield (Figure 1; *p* < 0.001). The acid-pepsin extract was also easier to filter than the acid-only extract. This improvement is attributable to pepsin’s specific cleavage of the telopeptide regions, which disrupts intermolecular cross-links without compromising the triple-helical backbone [34]. Importantly, removal of telopeptides not only increased yield but also likely exposed more side-chain functional groups (-COOH, -NH_2_, -OH) critical for subsequent electrostatic and hydrogen-bonding interactions with chitosan [27,28]. The higher viscosity of the non-pepsinized extract, due to residual cross-linked aggregates, accounts for the greater filtration resistance.

### 3.2. Characterization of Fetal Bovine Hide Collagen

#### 3.2.1. SDS-PAGE

**Figure 2:**
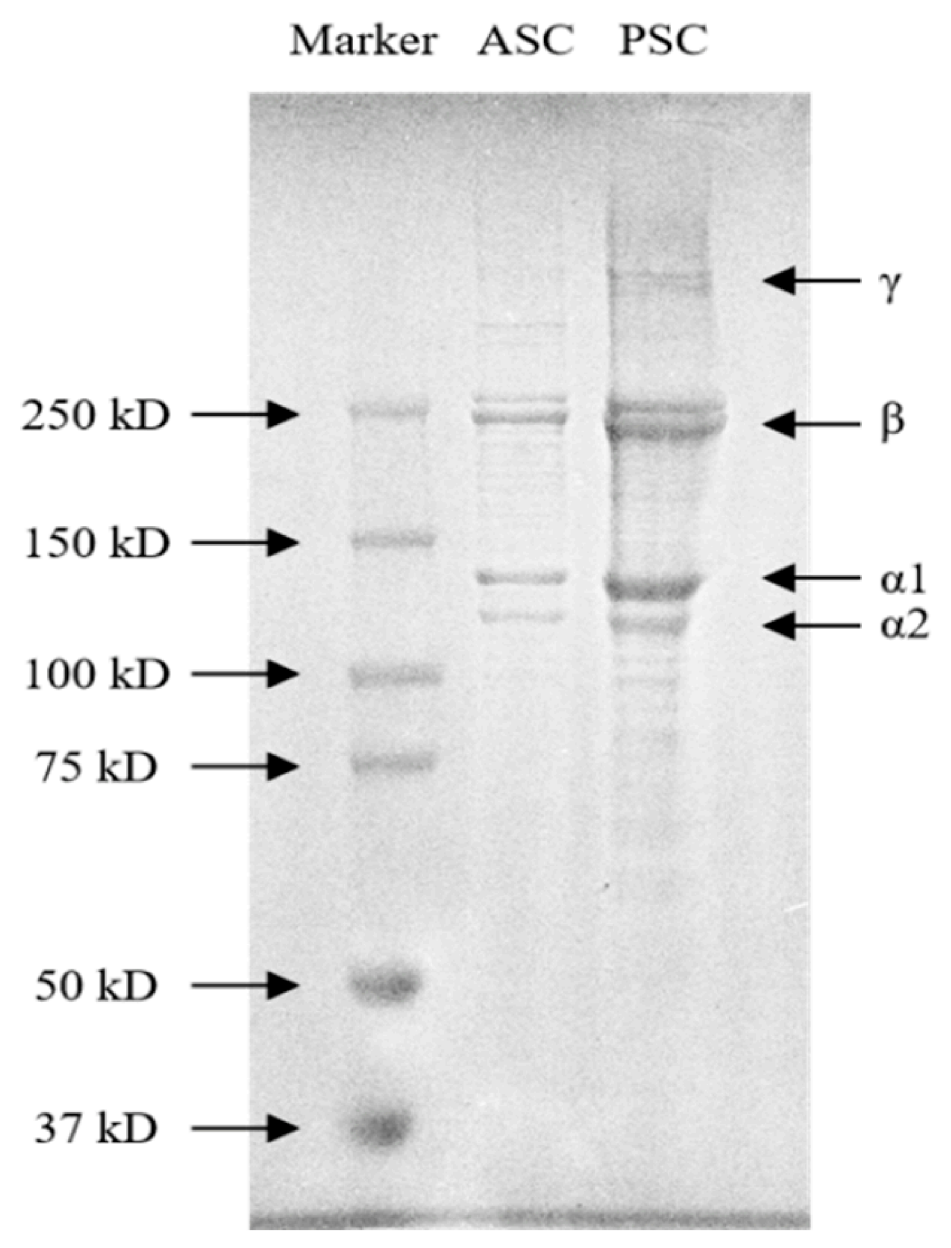
The SDS-PAGE pattern of ASC and PSC.

As shown in Figure 2, both ASC and PSC displayed the typical electrophoretic pattern of type I collagen, with γ, β, α_1_, and α_2_ chains. The molecular weights of α_1_ and α_2_ chains were approximately 130 and 110 kDa, consistent with type I collagen from camel [39] and bovine [40] sources. PSC showed only faint degradation bands below the α_2_ chain, which is characteristic of pepsin-mediated removal of non-helical telopeptides.

#### 3.2.2. UV-Vis Spectroscopy

**Figure 3:**
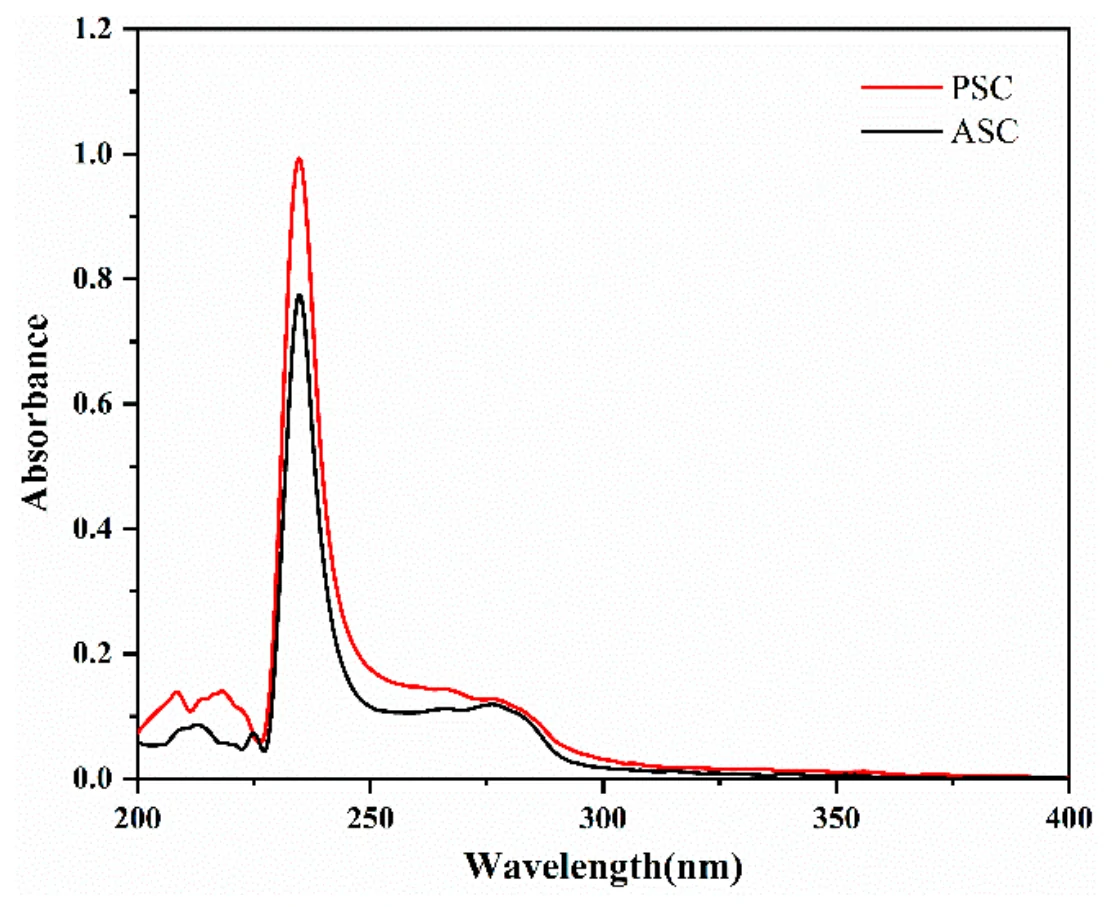
The ultraviolet absorption spectra of ASC and PSC.

Both ASC and PSC exhibited a maximum absorption peak at 234 nm (Figure 3), consistent with the typical feature of type I collagen [41]. ASC showed a weak shoulder at 280 nm, whereas PSC did not, indicating lower aromatic amino acid content and higher purity in PSC, which is beneficial for reducing immunogenicity [5].

#### 3.2.3. FTIR Spectroscopy

**Figure 4:**
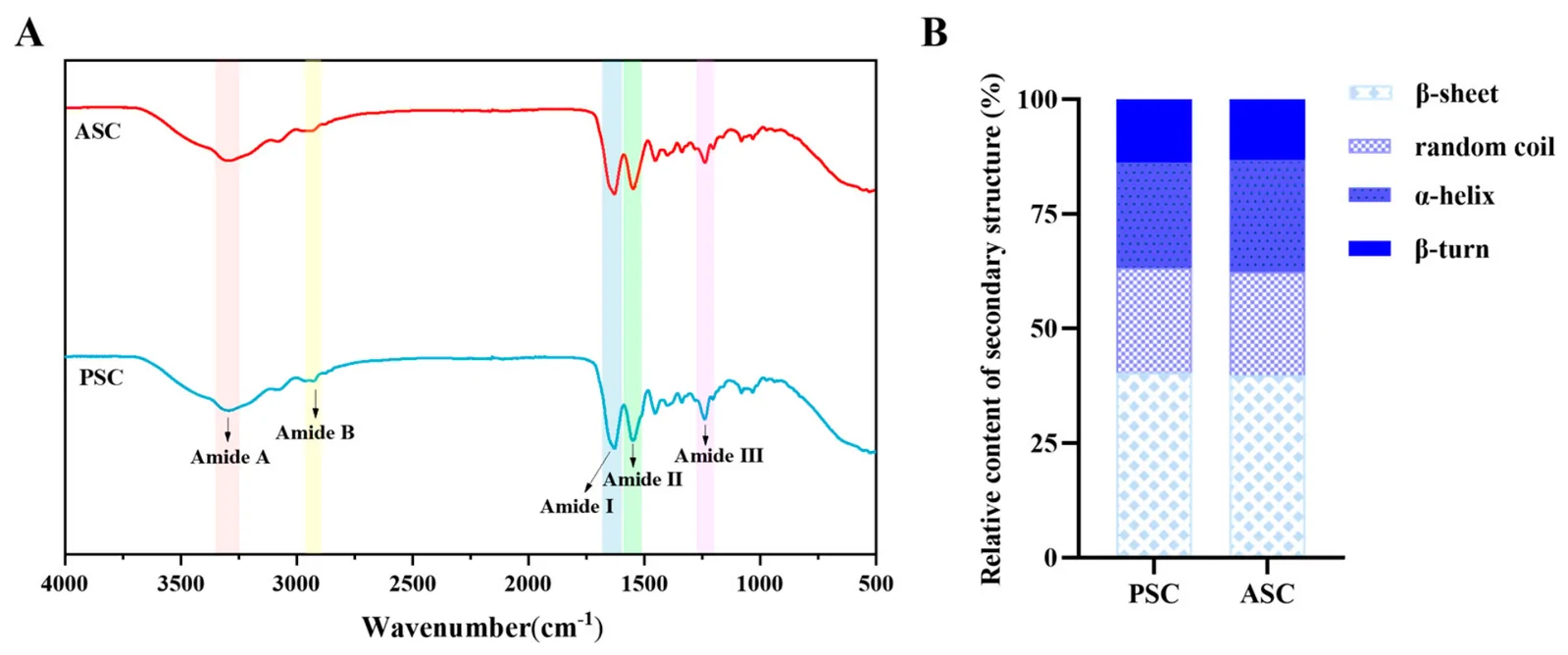
FTIR analysis of ASC and PSC. (**A**) FTIR spectra of ASC and PSC. (**B**) Relative contents of secondary structures of ASC and PSC determined from the amide I region.

The FTIR spectra (Figure 4A) of ASC and PSC showed characteristic amide A (~3293 cm^−1^), amide I (~1630 cm^−1^), amide II (~1550 cm^−1^), and amide III (~1238 cm^−1^) bands, confirming the triple-helical structure. Deconvolution of the amide I region (Figure 4B) revealed that acid extraction reduced the intensity of triple-helix characteristic peaks while increasing random coil signals, indicating effective disruption of hydrogen-bond networks. Pepsin treatment also caused subtle secondary structural changes [42,43]. These results collectively confirm that both extraction methods preserved the overall integrity of the triple helix.

#### 3.2.4. SEM Morphology

**Figure 5:**
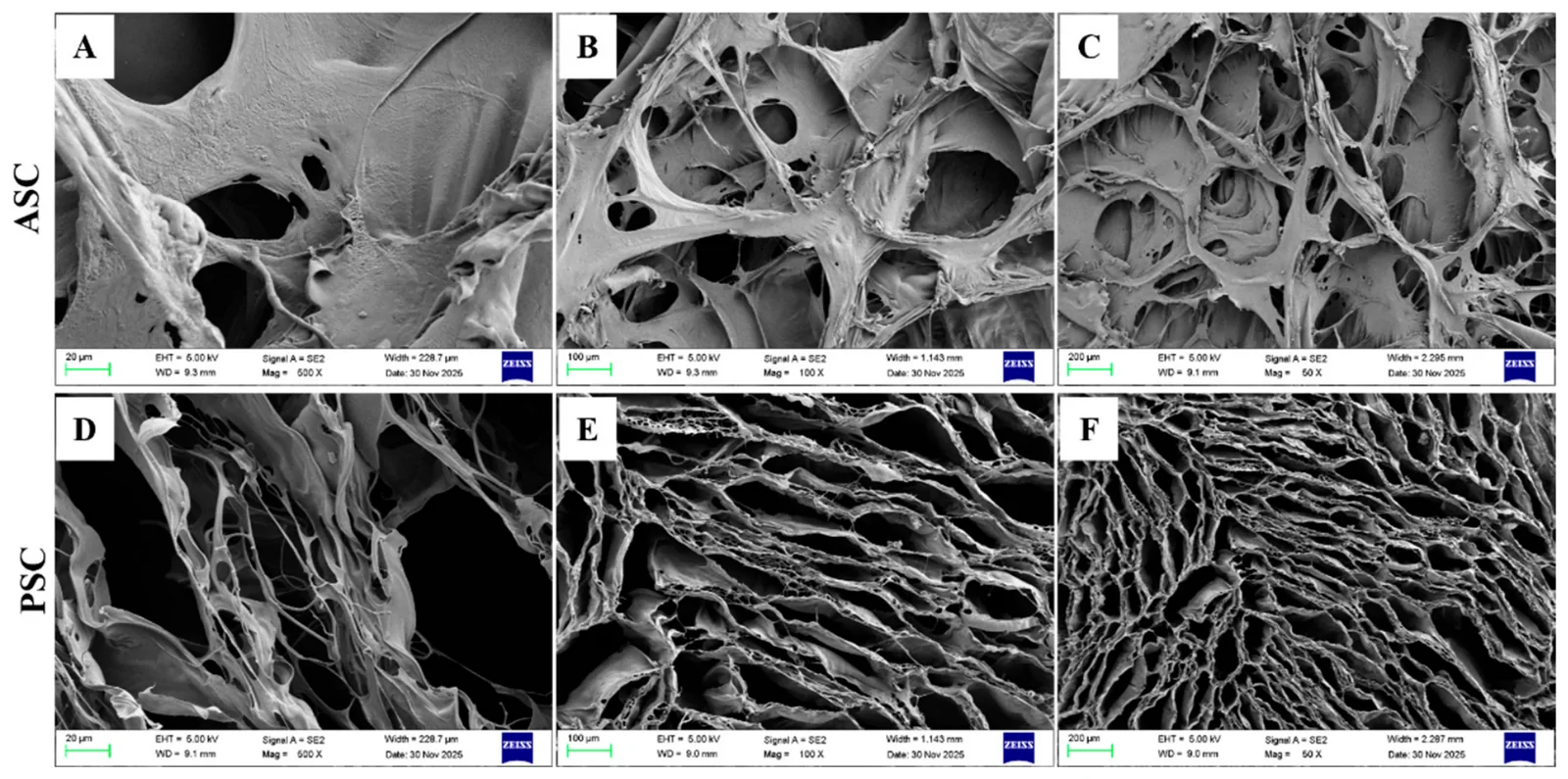
SEM images of (**A**–**C**) ASC and (**D**–**F**) PSC at magnifications of (**A**,**D**) 100×, (**B**,**E**) 500×, and (**C**,**F**) 1.0 K×.

SEM images (Figure 5) revealed marked morphological differences: ASC displayed densely packed, multilayered lamellar aggregates with a compact and uneven structure, whereas PSC exhibited a more porous, sheet-like morphology with a more uniform pore distribution. We propose that pepsin-mediated cleavage of covalent cross-links, particularly aldehyde condensation cross-links in the telopeptide region, reduces the propensity for ordered lateral aggregation, thereby facilitating the formation of an isotropic, open pore network during freeze-drying. In contrast, the retained telopeptide cross-links in ASC promote lateral aggregation, resulting in denser lamellar structures [28,34]. Thus, enzymatic modulation of collagen self-assembly offers a strategy for pre-designing sponge microstructure, providing a raw-material-level control for optimizing composite sponge performance.

### 3.3. Physicochemical Properties of COL/CS Composite Sponges

**Figure 6:**
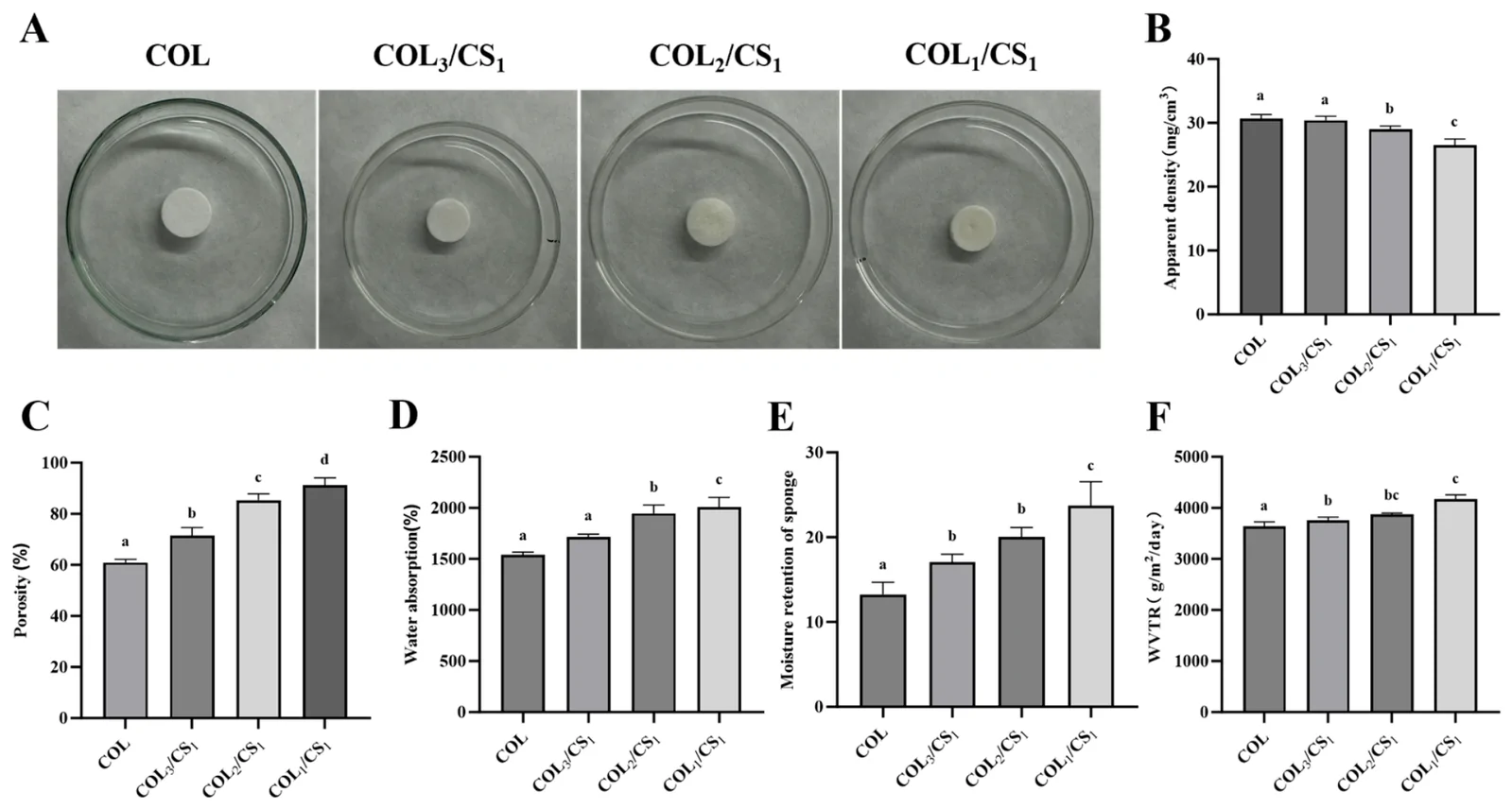
Physicochemical properties of the COL/CS composite sponges. (**A**) Collagen sponge apparent figure; (**B**) Apparent density of collagen sponge; (**C**) Porosity of collagen sponge; (**D**) Water absorption of collagen sponge; (**E**) Moisture retention of collagen sponges; (**F**) Water vapor transmission rate of collagen sponges. Different lowercase letters indicate significant differences (*p* < 0.05), while the same letters indicate no significant differences (*p* > 0.05).

Macroscopically (Figure 6A), all sponges were white, cylindrical, and velvety, with COL_1_/CS_1_ being the most regular. Apparent density (Figure 6B) decreased with increasing chitosan content, with COL_1_/CS_1_ showing the lowest value (26.52 ± 0.95 mg/cm^3^, *p* < 0.05). Porosity (Figure 6C) exhibited an opposite trend, with COL_1_/CS_1_ reaching the highest value (91.29 ± 2.82%, *p* < 0.05). Under fixed mold volume, a lower collagen content allows more ice crystal formation, leading to higher porosity after lyophilization. More importantly, the 1:1 ratio may achieve an optimal balance between electrostatic complexation and hydrogen-bonding networks [27,28,29]. The amino groups (-NH_3_^+^) of chitosan and carboxyl groups (-COO^−^) of collagen may form electrostatic complexes under weakly acidic to neutral conditions, while hydroxyl groups engage in intermolecular hydrogen bonding with carbonyl and amide groups. This multimodal interaction may inhibit excessive collagen aggregation, leading to a finer, more uniform, and better-connected pore network compared to pure collagen [29].

Water absorption (Figure 6D) and moisture retention (Figure 6E) were also preferred for COL_1_/CS_1_, reaching 2010 ± 94% and 23.70 ± 2.86%, respectively (*p* < 0.05). High porosity provides capillary action for rapid water uptake, while abundant hydrophilic groups on chitosan form strong hydrogen bonds with water molecules, retarding evaporation. This dual characteristic—rapid exudate absorption combined with maintenance of a moist wound environment—is essential for an ideal dressing [19,20]. Water vapor transmission rate (WVTR) (Figure 6F) was also highest for COL_1_/CS_1_ (4169.02 ± 86.45 g/m^2^/day), *p* < 0.05, facilitating timely removal of excess exudate and maintaining appropriate oxygen tension. Based on these comprehensive indices, COL_1_/CS_1_ was selected as the preferred formulation for subsequent evaluations.

### 3.4. In Vitro Hemocompatibility

**Figure 7:**
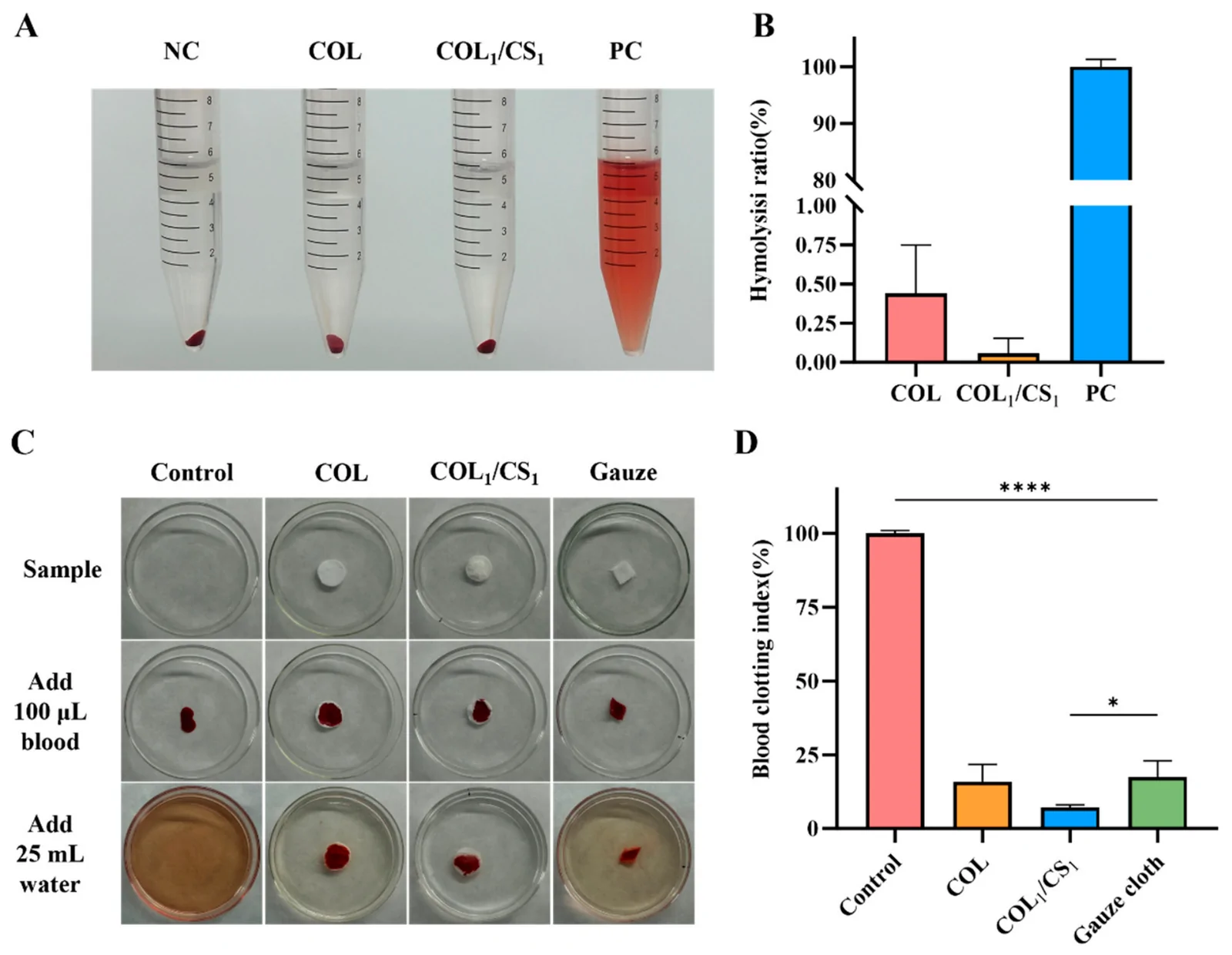
In vitro hemolysis and coagulation tests. (**A**) Schematic illustration of the hemolysis test; (**B**) Hemolysis ratio of each group; (**C**) Schematic illustration of the in vitro coagulation test; (**D**) BCI of each group (*n* = 3, * means *p* < 0.05, **** means *p* < 0.0001: significant differences compared to the control group).

Hemolysis results (Figure 7A,B) showed that both COL and COL_1_/CS_1_ groups had hemolysis rates below 5%, meeting the ISO 10993-4 requirements for medical devices [44,45]. In whole-blood coagulation tests (Figure 7C,D), the BCI of COL_1_/CS_1_ dropped to 7.02 ± 0.98%, significantly lower than that of COL (15.81 ± 5.91%) and gauze (17.49 ± 5.46%) (*p* < 0.05), indicating superior in vitro clotting performance.

Based on literature and material properties, we propose a possible triple synergistic mechanism for the enhanced pro-coagulant activity of COL_1_/CS_1_ [12,46,47]: (1) physical concentration—the high water absorption (2010%) rapidly concentrates clotting factors (e.g., fibrinogen, thrombin) and platelets at the wound site, accelerating the coagulation cascade; (2) electrostatic recruitment—the positively charged -NH_3_^+^ groups of chitosan actively attract negatively charged red blood cells and platelet surfaces, triggering pseudopod extension; (3) specific binding—we hypothesize that the intact GFOGER sequence in fetal bovine collagen may binds with high affinity to integrin α_2_β_1_ on platelets, potentially contributing to platelet adhesion, activation, and aggregation [10,11,12]. Pure collagen lacks the electrostatic recruitment function, and gauze possesses neither, hence the lowest BCI for COL_1_/CS_1_.

### 3.5. In Vitro Cytocompatibility

**Figure 8:**
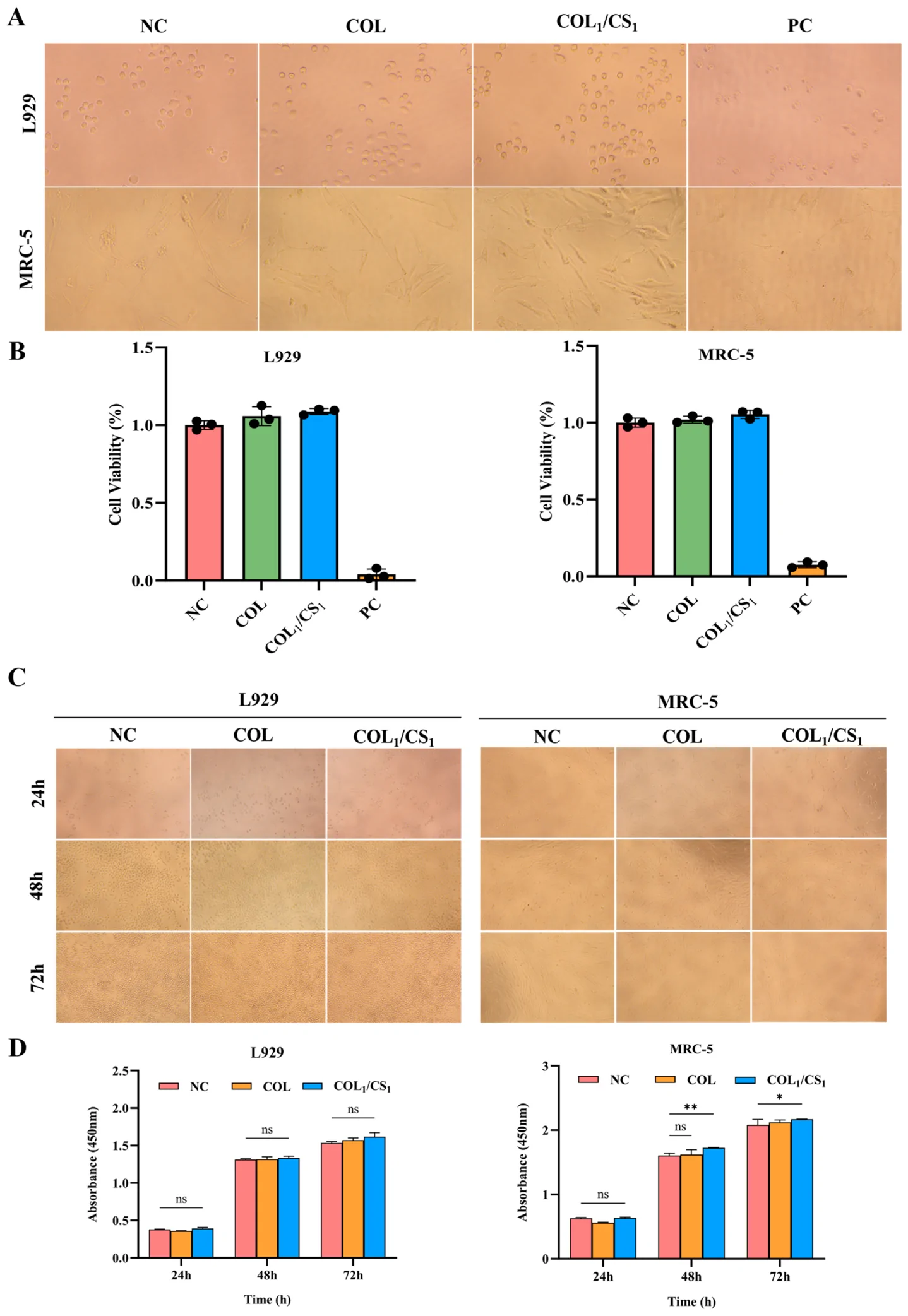
Cytocompatibility evaluation of COL and COL_1_/CS_1_ sponges. (**A**) Cell morphology of L929 and MRC-5 cells; (**B**) Cell viability of L929 and MRC-5 cells; (**C**) Proliferation morphology of L929 and MRC-5 cell; (**D**) The absorbance of L929 and MRC-5 cells cultured for different time. (*n* = 3, * means *p* < 0.05, ** means *p* < 0.01: significant differences compared to the negative control group).

CCK-8 results (Figure 8A,B) showed that after 24 h of culture with L929 and MRC-5 cells, the extracts of COL and COL_1_/CS_1_ both yielded cell viabilities above 100%, far exceeding the 70% cytotoxicity threshold specified in ISO 10993-5, indicating no cytotoxicity. Cell morphology (Figure 8C) showed no obvious pathological changes. Proliferation curves (Figure 8D) demonstrated continuous increases in OD values at all time points, surpassing the blank control, with COL_1_/CS_1_ maintaining a significant pro-proliferative effect on MRC-5 cells even at 72 h (*p* < 0.05).

Our results suggest that fetal bovine collagen/chitosan composites exert a stronger pro-fibroblast proliferative effect than pure collagen. This can be attributed to multiple mechanisms [10,24,29]: (1) topological cues—the more uniform and open pore network of COL_1_/CS_1_ provides better three-dimensional space and contact guidance for cells; (2) synergistic signaling—chitosan degradation products, such as chitooligosaccharides, may act as mild biological signals, upregulating proliferation-related gene expression in fibroblasts; (3) enhanced exposure of active motifs—complexation with chitosan may induce conformational changes in collagen fibrils, potentially exposing more integrin-binding sites (e.g., GFOGER) and thereby possibly activating downstream FAK-MAPK pathways [11].

### 3.6. Scratch Wound Healing Assay

**Figure 9:**
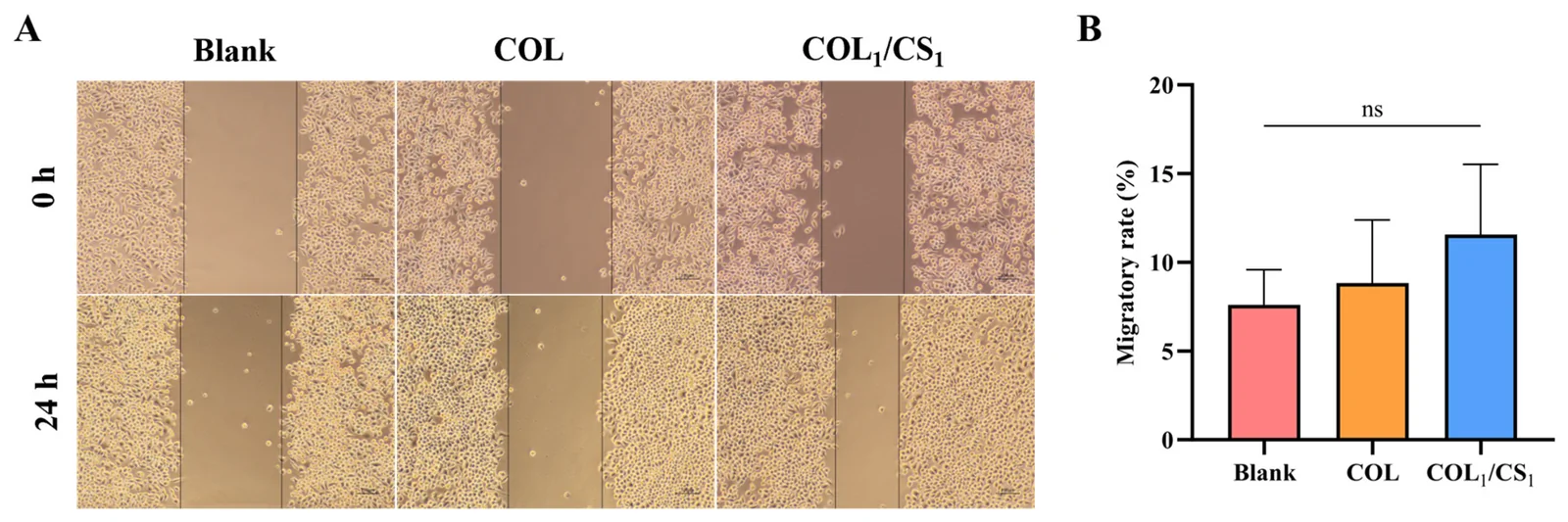
Cell migration assay of L929 fibroblasts. (**A**) Representative scratch wound images at 0 h and 24 h; (**B**) Quantitative analysis of scratch closure (*n* = 3; ns means not significant; significant differences compared to the blank control group).

The scratch assay (Figure 9) evaluated the effect of materials on L929 fibroblast migration. Compared with the control, both COL and COL_1_/CS_1_ groups promoted scratch closure within 24 h, with COL_1_/CS_1_ showing the strongest effect (Figure 9B). The introduction of chitosan further enhanced this effect, possibly through activation of Rho family GTPases (e.g., Rac1 and Cdc42), which play key roles in cytoskeletal reorganization and lamellipodia formation [24]. Enhanced wound repopulation facilitates extracellular matrix protein synthesis, contributing positively to accelerated wound repair.

### 3.7. In Vivo Wound Healing Efficacy

**Figure 10:**
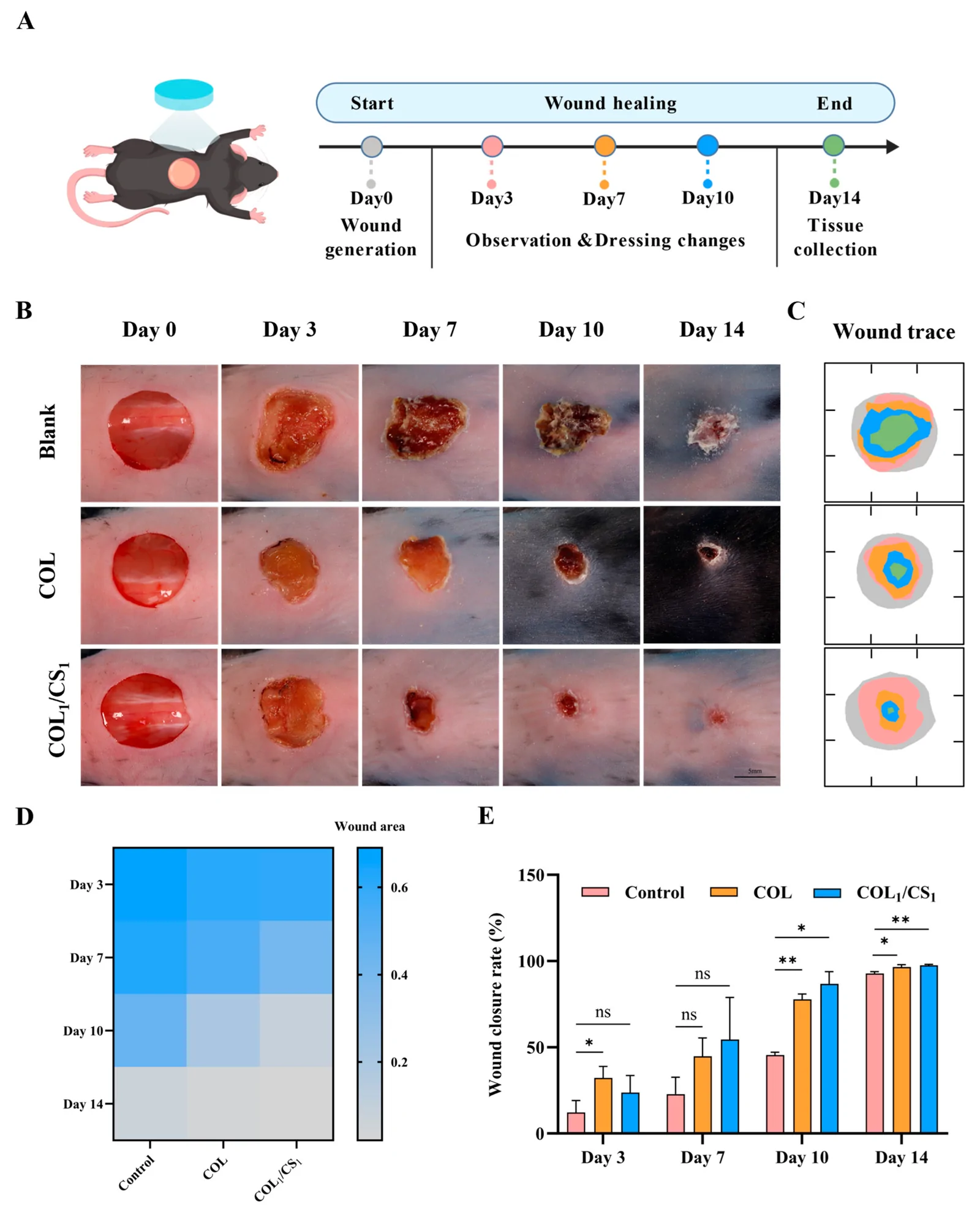
In vivo wound healing evaluation. (**A**) Animal experiment diagram; (**B**) Wound healing pictures at different time points; (**C**) Tracer diagram of residual wound area at different time points; (**D**) Heat map of wound area at different time points; (**E**) Statistical analysis of wound healing rate in mice at different time points (*n* = 4, * means *p* < 0.05, ** means *p* < 0.01: significant differences compared to the control group).

In the murine full-thickness defect model (Figure 10A), wound areas in all three groups decreased over time (Figure 10B,C). By day 14, the COL_1_/CS_1_ group achieved a wound closure rate of 98.10%, significantly higher than the COL and control groups (Figure 10D,E, *p* < 0.05), indicating the fastest wound closure capability.

An ideal dressing should dynamically match the different phases of wound healing. The COL_1_/CS_1_ sponge exhibited performance consistent with such adaptive behavior [19,20]: during hemostasis and inflammation (days 0–3), its high absorbency and pro-coagulant function rapidly controlled bleeding, while its porous structure provided a scaffold for inflammatory cell infiltration; during the proliferation phase (days 7–10), the hydrogel-like environment formed after absorption of exudate, combined with its pro-migration/pro-proliferation activities, effectively supported fibroblast ingrowth and angiogenesis; during the remodeling phase (day 14), the material guided orderly collagen deposition and remodeling. Pure collagen lacked the electrostatic recruitment and immunomodulatory functions of chitosan, while gauze served only as a physical cover, thus resulting in inferior healing compared to COL_1_/CS_1_.

### 3.8. Histopathological Analysis

**Figure 11:**
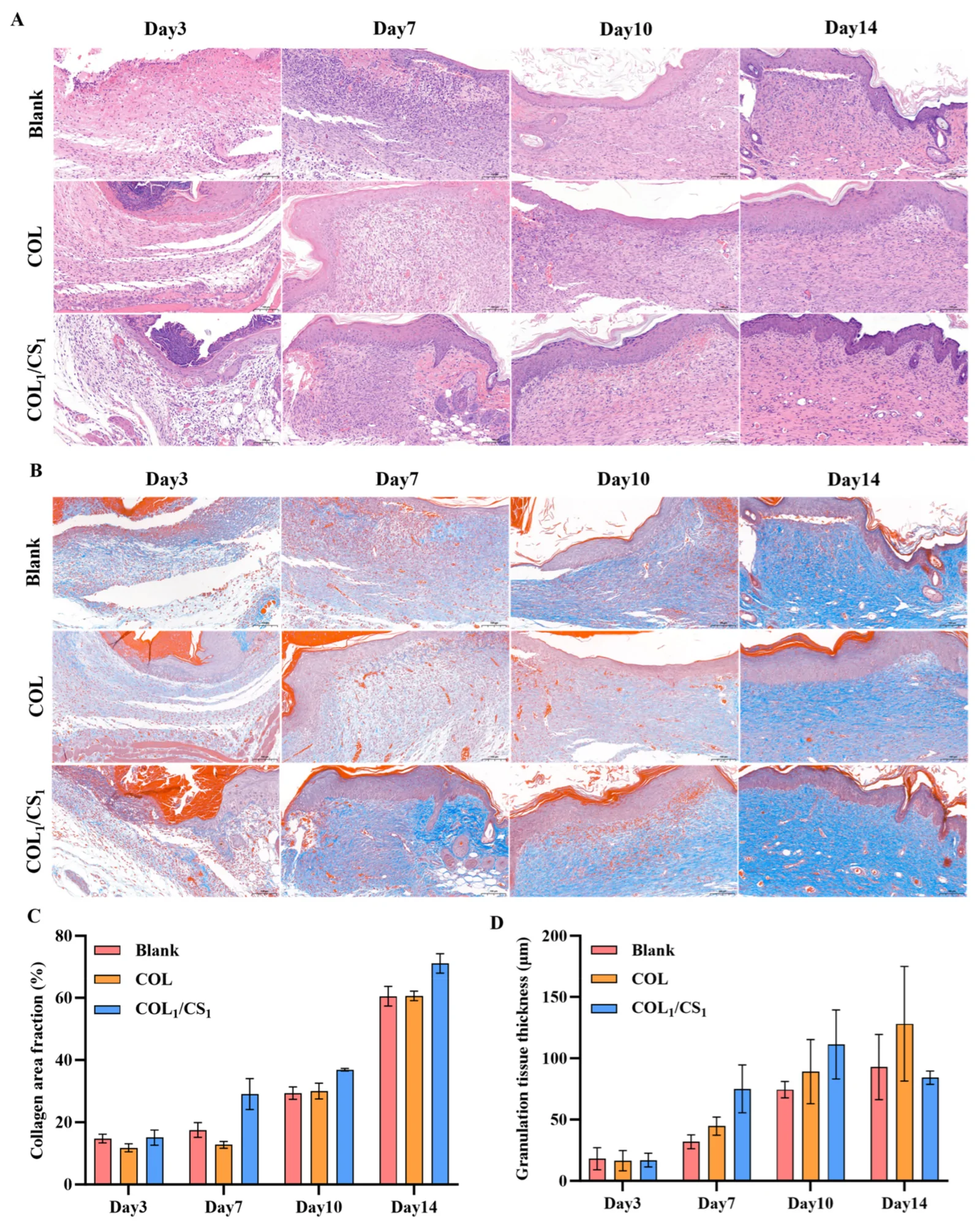
Histological evaluation of skin wound healing in mice. (**A**) H&E staining of mouse skin tissue. (**B**) Masson staining of mouse skin tissue. (**C**) Collagen area fraction. (**D**) Granulation tissue thickness.

H&E staining (Figure 11A) revealed that on day 7 post-surgery, the COL_1_/CS_1_ group already showed interwoven fibroblasts and collagen fibers, along with neovascularization and epithelial tissue formation. By day 10, granulation tissue was dense, and the epithelial layer was nearly continuous. On day 14, the granulation tissue was more mature, smoothly transitioning to surrounding normal tissue, with orderly cell arrangement and densely packed, well-organized collagen, closely resembling normal skin.

Masson’s trichrome staining (Figure 11B) assessed collagen deposition. On day 7, the COL_1_/CS_1_ group exhibited denser and more uniformly distributed blue-stained collagen fibers. By day 10, collagen fibers aligned parallel to the epithelial layer with clear tissue stratification. On day 14, the collagen fibers were the densest and most orderly, achieving excellent tissue remodeling close to normal tissue.

Collagen area fraction analysis (Figure 11C) revealed that the COL_1_/CS_1_ group exhibited higher collagen deposition than the Blank and COL groups at all time points, reaching 71.12% ± 3.14% by day 14, which was consistent with the Masson staining observations and indicated that the COL_1_/CS_1_ composite sponge effectively promoted collagen deposition and remodeling during wound healing. Granulation tissue thickness measurement (Figure 11D) showed that the Blank and COL groups continuously increased throughout the observation period, reaching 92.87 ± 26.55 μm and 128.13 ± 46.75 μm, respectively, by day 14, which were markedly greater than normal skin thickness. In contrast, the COL_1_/CS_1_ group peaked at day 10 (111.30 ± 28.19 μm) and then declined to 84.20 ± 5.36 μm by day 14, approaching the thickness of surrounding normal skin.

These results indicate that COL_1_/CS_1_ not only accelerates healing speed but also improves healing quality [24,48]. The biphasic pattern of granulation tissue thickness in the COL_1_/CS_1_ group—increasing during the proliferative phase and declining during the remodeling phase—is consistent with the physiological progression of normal wound healing, suggesting that the composite sponge effectively facilitated the orderly transition from proliferation to remodeling. In contrast, the sustained thickening observed in the Blank and COL groups indicates delayed healing that had not yet entered the remodeling phase. The disorganized collagen fibers in the control group suggested scar-prone healing, whereas the COL_1_/CS_1_ group achieved functional regeneration. This is attributed to the low immunogenicity of fetal bovine collagen and the immunomodulatory function of chitosan—the latter possibly promoting macrophage polarization toward the M2 (pro-regenerative) phenotype [25].

### 3.9. In Vivo Organ Toxicity

**Figure 12:**
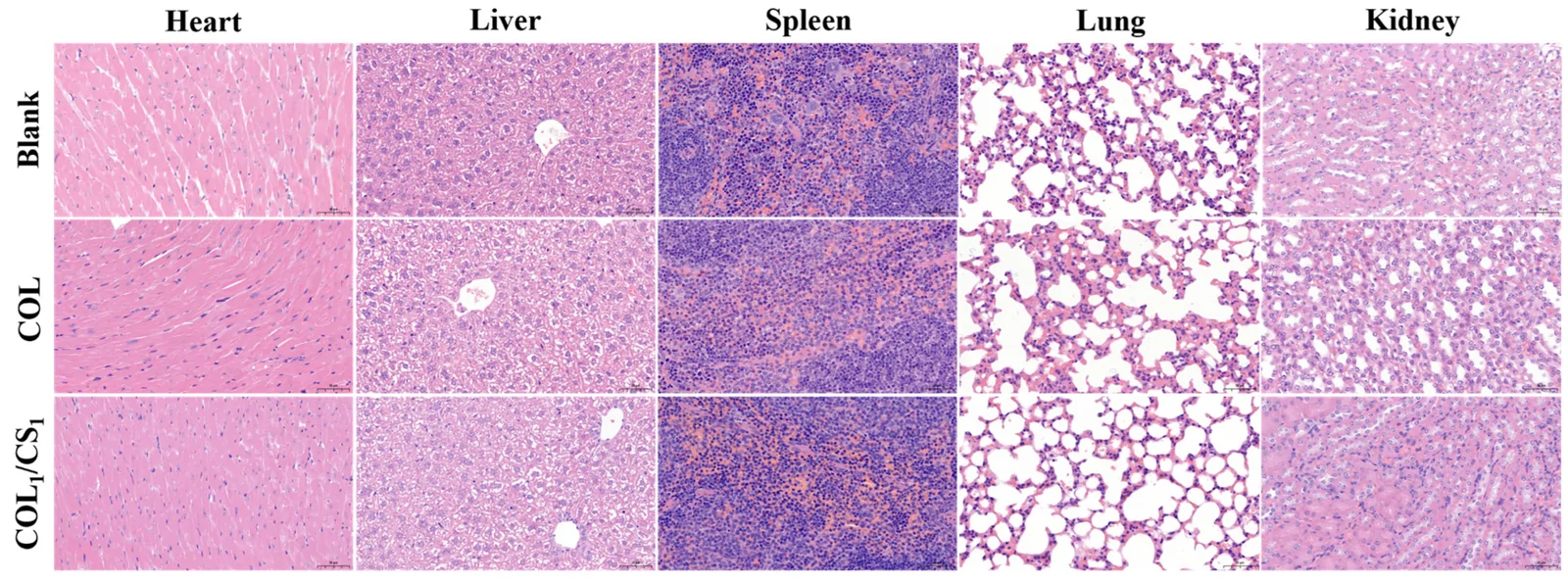
H&E staining of main organs and tissues of mice on the 14th day.

On day 14, H&E staining of heart, liver, spleen, lung, and kidney (Figure 12) showed no obvious pathological changes in the COL and COL_1_/CS_1_ groups compared to the control. Cardiomyocytes were regularly arranged without inflammatory infiltration; liver lobules were normal with no necrosis; spleen showed abundant lymphocytes; alveolar structures were intact; and glomeruli and renal tubules were clearly defined. The absence of pathological alterations in major organs suggests that the COL_1_/CS_1_ sponge does not induce overt acute systemic toxicity under the present experimental conditions [44]. This finding is limited to the 14-day observation period and does not confirm long-term biosafety or safety in compromised physiological states.

### 3.10. Limitations and Future Directions

We acknowledge several limitations of the present study. First, we did not include collagen from adult bovine or other sources as a direct comparator; therefore, the attribution of the observed benefits specifically to the fetal origin remains a plausible hypothesis rather than a demonstrated conclusion. Second, the proposed molecular mechanisms—including GFOGER–integrin interactions, FAK/MAPK signaling, Rho GTPase activation, and M2 macrophage polarization—were not directly measured; these explanations are based on literature and should be considered speculative until further experimental validation. Third, we did not perform mechanical testing (e.g., wet-state compressive strength) or degradation studies, which are important for practical applications; the sponge’s handling integrity was satisfactory, but quantitative data are lacking. Fourth, the in vitro assays were performed using material extracts, which cannot capture the topographical or contact-guidance effects of the three-dimensional scaffold architecture. Fifth, the in vivo evaluation was conducted in an acute healthy wound model over 14 days; long-term safety, chronic wounds, and infected wounds remain to be tested. Finally, antibacterial activity, although a known property of chitosan, was not evaluated. These limitations should be addressed in future work to further substantiate the translational potential of this material.

## 4. Conclusions

In this study, we successfully extracted type I collagen from fetal bovine hide—a major byproduct of the fetal bovine serum industry—and constructed a series of collagen/chitosan composite sponges. The best-balanced COL_1_/CS_1_ sponge exhibited excellent physicochemical properties, good hemocompatibility and cytocompatibility, and a marked ability to promote fibroblast repopulation (as indicated by scratch closure). In a murine full-thickness skin defect model, COL_1_/CS_1_ significantly accelerated wound closure, promoted orderly granulation tissue deposition, enhanced collagen remodeling and neovascularization, and displayed no overt toxicity in major organs under the tested conditions. From a molecular mechanistic perspective, we hypothesize that the lower cross-linking density of fetal bovine collagen may enhance electrostatic and hydrogen-bonding interactions with chitosan, yielding a more uniform porous structure that supports cellular behavior and facilitates rapid hemostasis. However, this hypothesis requires direct confirmation through comparative studies with adult collagen and through molecular signaling analysis. This work provides a viable strategy for valorizing an agricultural byproduct into high-value biomedical materials and offers insights into the potential importance of collagen source-dependent properties in biomaterial performance. Future studies should provide direct molecular evidence (e.g., integrin signaling pathways), evaluate mechanical properties and degradation behavior, and validate the efficacy in clinically relevant models such as infected or diabetic wounds.

## Figures and Tables

**Table 1:** Composition of collagen–chitosan composite sponge.

Sample Designation	CS Content (%)
COL	0
COL_3_/CS_1_	25
COL_2_/CS_1_	33
COL_1_/CS_1_	50

## Data Availability

The original contributions presented in the study are included in the article, further inquiries can be directed to the corresponding author.
